# Empirical investigation of e-health intervention in cervical cancer screening: A systematic literature review

**DOI:** 10.1371/journal.pone.0273375

**Published:** 2022-08-19

**Authors:** Rodziah Romli, Rahana Abd Rahman, Kah Teik Chew, Syahnaz Mohd Hashim, Emma Mirza Wati Mohamad, Azmawati Mohammed Nawi

**Affiliations:** 1 Department of Community Health, Faculty of Medicine, Universiti Kebangsaan Malaysia, Cheras, Kuala Lumpur, Malaysia; 2 Institut Latihan Kementerian Kesihatan Malaysia Alor Setar, Ministry of Health, Putrajaya, Malaysia; 3 Department of Obstetrics and Gynaecology, Faculty of Medicine, Universiti Kebangsaan Malaysia, Cheras, Kuala Lumpur, Malaysia; 4 Department of Family Medicine, Faculty of Medicine, Universiti Kebangsaan Malaysia, Cheras, Kuala Lumpur, Malaysia; 5 Centre for Research in Media and Communication (MENTION), Faculty of Social Sciences and Humanities, Universiti Kebangsaan Malaysia, Bangi, Selangor, Malaysia; SRM Institute of Science and Technology, INDIA

## Abstract

Cervical cancer (CC) screening can detect the cancer early but is underutilized, especially among the developing countries and low- to middle-income countries. Electronic health (e-health) has the potential for disseminating health education and is widely used in the developed countries. This systematic literature review investigates the effectiveness of e-health intervention for improving knowledge of CC and the intention or uptake for CC screening. We followed the PRISMA 2020 guideline and registered with PROSPERO (registration ID CRD42021276036). We searched the Web of Science, Scopus and EBSCO Medline Complete databases for eligible studies. Studies that conveyed informational material through e-health intervention were selected. The results were analyzed using narrative synthesis, and the pooled estimates were calculated using meta-analysis. A total of six studies involving 1886 women were included in this review. The use of e-health aids alone led to increased knowledge. The meta-analysis demonstrated that the mixed-education method of e-health movies and video education with didactic sessions increased CC screening uptake. A random-effects model revealed that CC screening uptake following e-health interventions were almost double of that of their comparison (odds ratio = 2.29, 95% confidence interval: 1.28–4.10, p < 0.05). Various areas of study demonstrated e-health intervention effectiveness (minority communities, urban areas, rural areas). Health education through e-health intervention has huge potential for promoting CC screening in the community. Nevertheless, the use of appropriate frameworks, user engagement and culturally tailored e-health need to be prioritized.

## Introduction

Cervical cancer (CC) remains a burden and a global issue. CC is a slowly progressing disease, starting as an intraepithelial lesion that takes up to 10 years to develop into a precancerous lesion [1, 2]. Early-stage CC is usually asymptomatic, leading to patients being unaware of it [1]. Vaccination is about 90% effective in preventing CC related to HPV type 16 and 18 [1], but secondary screening programs such as Pap smear should be continued for early detection of cervical cell changes [2]. A recent study demonstrates low knowledge among HPV vaccinated females and they do not consider regular Pap smears as an important CC screening tool following HPV vaccination [3]. Furthermore, a review paper that compare knowledge on HPV infection, CC and HPV vaccines among young women, concluded the level of knowledge was higher among developed countries but is still insufficient [4]. There is evidence of avoidance among developing country due to the high cost of the vaccine [5], whereas, low incidence of CC is perceived to be linked to the low HPV vaccination in developed country [6]. The success of reducing CC cases depend on screening strategies in detecting and treating most of the precursors of CC before it becomes invasive, as well as mass vaccination being implemented among adolescents [7].

Developed countries with effective screening methods have successfully reduced CC cases by up to 80% [8, 9]. However, almost 90% of deaths occurs in the developing countries and low- to middle-income countries (LMICs), which are less successful in implementing screening methods [1, 2, 9]. Currently, the developed countries use electronic health (e-health) interventions and media campaigns as an education method as a strategy to reach more women to promote CC screening [10–13]. Therefore, investing in e-health promotion programs might enable the cultivation of knowledge on the benefits of CC screening and promote lifelong screening for preventing CC.

Recently, the World Health Organization (WHO) asserted that CC prevention and early diagnosis are highly cost-effective with the use of existing technology and tools [8]. The Internet of Things is defined as a network that connects devices to the internet to measure health data, which leads to quality low-cost medical care. This has led to e-health becoming a medium with tremendous potential for conveying health information to society [14]. Apart from e-health, mobile health (m-health) is also been used as a medium for disseminating information using digital technology. The e-health medium covers wide range of technologies including computers, telephony and wireless communication, whereas the m-health was a part of e-health that deliver services via mobile and wireless technology [15].

E-health intervention conveys informational materials in various formats, such as audio-visual aids, educational videos, telefilms, small media or interactive multimedia programs. However, there is a risk of failure to involve disadvantaged groups such as the elderly, those with lower education and those living in rural areas [16]. The ability to make use of e-health technology and be self-reliant allows one to be directly involved with health management [17]. People who are e-health literate can access enhanced healthcare services, whereas those who are not literate or knowledgeable about e-health cannot [17]. Therefore, e-health literacy might also contribute to the effectiveness of e-health intended outcome.

The present systematic literature review is aimed at assessing the effectiveness of e-health intervention for improving knowledge of CC and the intention or uptake for CC screening. In addition, e-health intervention use through different areas and setting was also evaluated. This review may help identify future practice and strategies for improving CC screening worldwide.

## Methods

### Protocol registration

The review was part of a clinical research approved by the Universiti Kebangsaan Malaysia Medical Research Ethics Committee (protocol code: FF-2021-499; date of approval: 29 October 2021). This systematic literature review and meta-analysis followed the PRISMA (Preferred Reporting Items for Systematic literature reviews and Meta-Analyses) 2020 guideline [18] and has been registered with PROSPERO (registration ID CRD42021276036, https://www.crd.york.ac.uk/prospero/display_record.php?ID=CRD42021276036).

### Eligibility criteria

The review criteria were developed based on PICO (Problem, Intervention, Comparison, Outcome). Problem referred to CC screening, which remains a global problem and is the fourth highest killer of women worldwide [19]. Intervention referred to e-health intervention that delivered via video education, informative videos, digital media or e-health videos. Comparison referred to alternative approach or conventional methods or any type of control group. The outcome of interest in the selected studies were knowledge on CC and intention or uptake towards CC screening.

The inclusion criteria of eligible study included the using of e-health as the intervention for disseminating information regarding CC and CC screening. Only type of study design of randomized controlled trials, non-randomized trials, quasi experimental such as pre and post study included in the review. The exclusion criteria included non-CC screening such as HPV vaccination, review article or case study.

### Information sources and search strategy

We searched for eligible studies on the Web of Science (WoS), Scopus and EBSCO Medline Complete medical databases These databases have many relevant studies, with numerous open-access full-text articles available online. WoS has information analysis resources [20] and uses a subject area categorisation scheme [21], which helps in finding the right articles. Scopus has enhanced utility for medical literature research, which includes a more expanded spectrum of journals [22]. Therefore, the selection of WoS, Scopus and EBSCO Medline Complete justified to covers an extensive range of medical subjects suitable for current updates related to the medical field and e-health intervention use.

Two Universiti Kebangsaan Malaysia public health physicians performed an initial screening to identify the potential keywords to determine whether this review was feasible. This search criteria were developed with support of an experience librarian in the faculty. The verified and validated medical subject heading (MeSH) keywords were: (“cervical cancer” OR “uterine cervical neoplasms” OR “uterine cervical carcinoma” OR “cervical tumor” OR “cervical malignancy” OR “cervix tumor” OR”cervix malignancy” OR “cervix cancer” OR “cervical neck tumor” OR “cervical neck malignancy” OR “cervical neck cancer” OR “uterine cervix cancer” OR “uterine cervix tumor” OR “uterine cervix malignancy” OR “cervix uteri cancer” OR “cervix uteri malignancy” OR “cervix uteri tumor”) AND (“screening” OR “Papanicolaou test” OR “Papanicolaou smear” OR “Pap smear” OR “Pap test” OR “human papillomavirus DNA tests” OR “HPV DNA tests” OR “human papillomavirus test” OR “HPV test”) AND (“electronic health” OR “electronic health intervention” OR “e-health” OR “video education” OR “informative video” OR “media” OR “digital media” OR “digital literacy”) (S1 Table).

We added potential studies through citation search of systematic reviews to enrich the literature search. The literature search was conducted from September 2021 to October 2021. The screening strategy was limited to articles from January 2011 to October 2021 and that had been published in English with full text available. The evolution of the e-health era among the developed countries began in 2006 [23] but it remains challenging for LMICs [24]. Current technology has been used widely to improve medical decisions by providing educational materials combined with multimedia materials, tailored education and decision support tools [25]. Thus, the 10-year publication span is justified.

### Selection process

**Fig 1:**
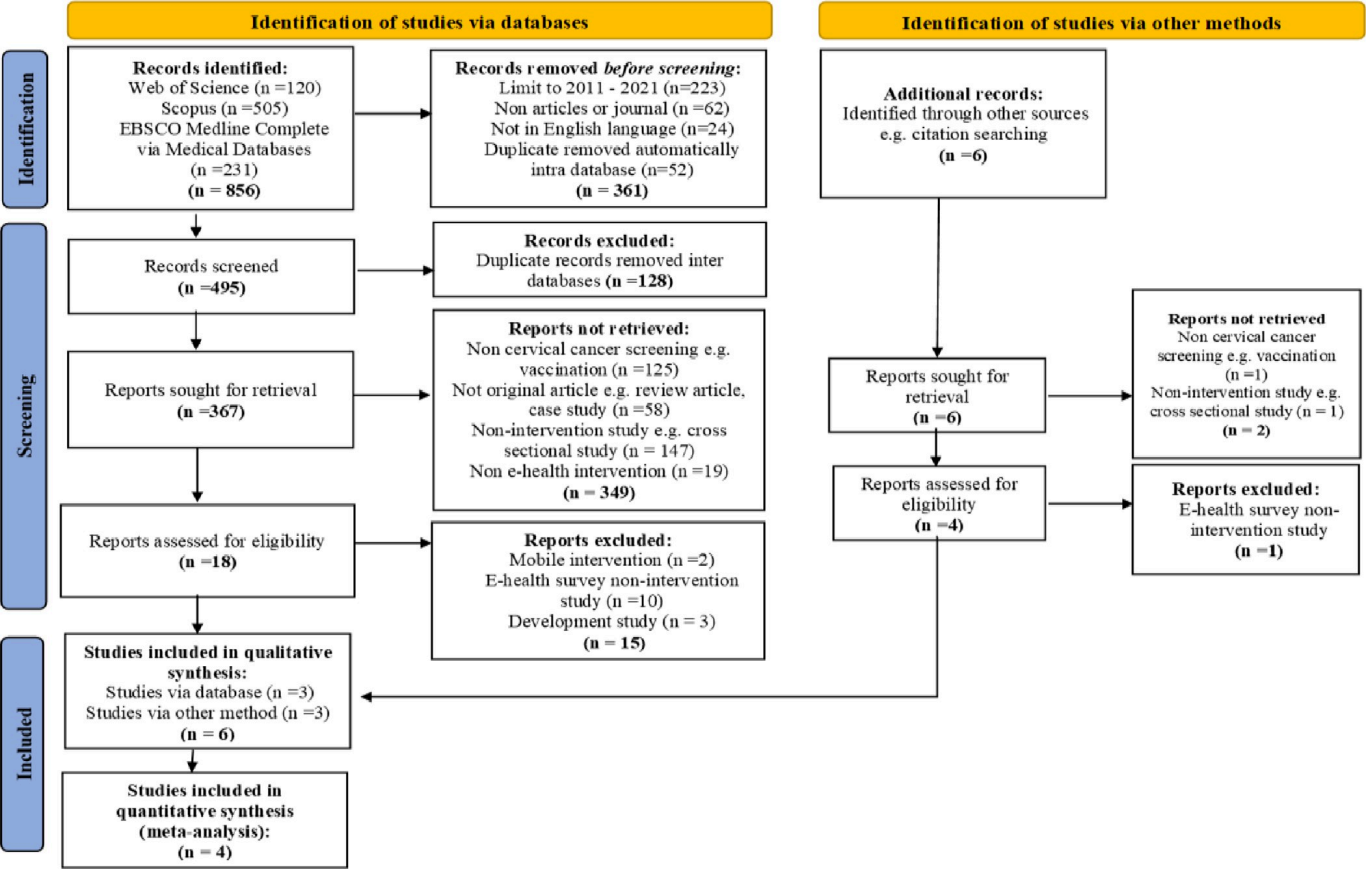
Selection process of study according to PRISMA 2020 guideline.

Initially, we retrieved 862 records (WoS = 120, Scopus = 505, EBSCO Medline Complete = 231, citation search = 6); 361 records were removed before screening (Fig 1). All data were imported into EndNote reference manager and duplicates were removed (n = 128). One author (RR) conducted individual evaluation of the titles and abstracts (n = 373) to identify potential reports based on the review inclusion and exclusion criteria. Three hundred and fifty-one reports were not eligible because they were non-CC screening articles, which was focusing on HPV vaccination (n = 126); non-original articles such as review article, case study (n = 58); non-intervention studies such as cross sectional study (n = 148) and non-e-health intervention studies (n = 19).

A total of 22 full-text articles were retrieved and their eligibility information was extracted to a Microsoft Excel sheet. Sixteen articles did not fulfil the inclusion criteria and were excluded due to a mobile intervention (n = 2), an e-health survey with no intervention (n = 11) and a development studies (n = 3). Both mobile (m) interventions were excluded from this review due to non-intervention study design. One study focus on development of mobile application-based learning [26]. Another study regarding the non-health education intervention which only focusing on online family group conversation [27]. The inclusion criteria were randomized, quasi-experiment or pre-test and post-test design studies.

A final six articles were selected for data extraction and quality assessment based on the research questions. Two authors (RR and AMN) reviewed the included studies independently. Discrepancies were resolved through discussion and consensus.

### Study risk of bias assessment

The study quality was appraised using the Effective Public Health Practice Project Quality Assessment Tool (EPHPP). The EPHPP has been considered an established, valid and reliable instrument for most current methods of systematic reviews of public health-related effectiveness since 1998 [28] and has fair inter-rater agreement for individual component ratings and excellent agreement for the final rating [29]. Bias was assessed in six component ratings, where the answers led to a global and final rating of ‘strong’, ‘moderate’ or ‘weak’. Two authors (RR and AMN) reviewed all selected articles independently and reached agreement for the final rating (S2 Table).

### Synthesis methods

All statistical data were synthesized using Review Manager 5.4 (Cochrane, London, UK) [30]. Data regarding mean knowledge score of CC, intention or uptake towards CC screening were extracted from each study. The odds ratios (ORs) with 95% confidence intervals (CIs) for dichotomous data (intention or uptake) were used as the effect measure and were reported. While the effect measure for continuous data (mean knowledge) was analysed using the mean difference of knowledge score and were reported. The continuous data, which measures on different scales outcome, can be combined using the effect size [31]. The larger the effect size the stronger the relationship between two variables (small:0.2; medium:0.5; large:0.8) [32]. Thus, we convert OR (dichotomous data) using Effect Size Converter [33] and mean difference (continuous data) using Practical Meta-Analysis Effect Size Calculator. We used the I^2^ statistic for each pooled estimate to assess inter-study heterogeneity and used a random-effects model for heterogeneity (p < 0.05). An I^2^ value of 25%, 50% and 75% indicates low, moderate and high heterogeneity, respectively [34]. Due to the possibility of clinical homogeneity, we performed subgroup analysis of the use of e-health aids alone and the screening uptake results only. Publication bias was assessed using funnel plots. The absence of heterogeneity indicated by 95% of the included studies lay within the boundaries of the funnel plot [35].

## Results

### Study characteristics

Table 1 provides an overview of the characteristics of the included studies. A total of six studies were included in this review and represented various ranges of estimated age-standardized incidence rates (ASR) of cervix uteri carcinoma from the International Agency of Research on Cancer GLOBOCAN 2022 [36]. Three studies conducted in the US, a developed country, had ASR of < 7.0 per 100 000 [11, 37, 38]. Two studies from African LMICs, i.e. Tanzania [39] had the highest ASR of ≥25.2 per 100 000 and Nigeria [40] with ASR between 16.7 to 25.2 per 100 000. A study conducted in India, a developing country, reported an ASR of 16.7 to 25.2 per 100 000 [41]. Three studies used e-health aids alone as their tool [11, 37, 39]. There were two studies from rural area [40, 41], a study from urban area [38], two studies related to minorities [11, 37] and a study comparing rural to urban [39]. Various areas of study showed the effectiveness for increasing screening intention and uptake, such as those on refugees [37] and women living in urban areas [38] and rural areas [40].

**Table 1:** Characteristics of studies in this review (n = 6).

No	Authors (Year)	Country (Targeted population)	Study Design	Intervention	Content of E-health	Outcome & Key Findings
**1**	Nagamma et al. 2020	India Rural area	Quasi-experimental	Type: Mix education method Duration: One day session [Total 7 sessions (18–25 participants each) within 1 year window] Tools: • IG: Audio-visual aid + 30 min face to-face interactive teaching session (n = 82) • CG: Pamphlet (n = 84)	Not mention	Knowledge of CC risk factor (pre to post-test): • IG: 6.4% to 79.2% • CG: 6.0% to 58.4% Knowledge regarding CC screening (pre to post-test): • IG: 30.4% to 100% • CG: 21.4% to 84.5%
**2**	Abiodun et al 2014	Nigeria Rural area	Quasi-experimental	Type: Mix Education method Duration: 4 hours session [Total 14 sessions (50 participants each) within 7 days] & 13 weeks post-intervention follow up. Tools: • IG: 25 min Health Education Movie “ASUNLE” + Didactic lectures + QA session + hand bill to be read at home (pre: n = 350; post: n = 325) • CG: Education on breast cancer & screening; didactic lectures (pre n = 350; post n = 289) • Both CG & IG received the other intervention after 13 weeks follow up assessment.	Not mention	Mean knowledge scores on CC (p<0.0001) • IG: pre-test (1.75 ± 5.65) post-test (25.69 ± 6.20) • CG: pre-test (2.03 ± 5.77) post-test (2.22 ± 6.04) Mean perception scores on CC screening • IG: pre-test (1.13 ± 0.77) post-test (4.43 ± 0.92) • CG: pre-test (1.16 ± 0.83) post-test (1.17 ± 0.88) Uptake of CC screening (pre-test to post-test): • IG: 4.3% to 8.3% • CG: 3.4% to 3.8% Intention to CC screening (pre-test to post-test): • IG: 89.7% to 92.3% • CG: 91.4% to 93.4%
**3**	Kessler et al. 2012	USA Urban area	Pretest and post-test prospective design.	Type: Mix education method Duration: One-day education program & 15 months post-intervention follow up. Tools: • Video on Pap tests & mammogram + Self-efficacy–based educational intervention (pre-test: n = 56; follow up: n = 47) *Study also included mammography screening. However, the result not been retrieved in this SLR.	Videos on mammograms and Pap tests that demonstrated the success of those procedures through vicarious experiences.	Mean knowledge scores [Breast and Cervical Health (BACH) survey] • pre-test: 7.94 ±1.41 • post-test: 8.89 ±0.6 • follow up: 8.74 ±0.79 Uptake of CC screening after 15 months • pre-test: 70% follow up: 85%
4	Cooper et al. 2021	Africa Rural vs Urban	Pretest and post-test prospective design.	Type: E-health aid Duration: Daily VIA screen-and-treat workshops (Total 5 days) Tools: 15 min MedicalAidFilms (n = 764)	Understanding screening, treatment, and prevention of cervical cancer.	Mean knowledge score (p<0.0001) • Pre-test: 2.22 ±1.76 • Post-test: 3.86 ±1.78 • Urban area: 4.54 ± 1.56 • Rural area: 2.78 ± 1.57 Significantly improved regardless age group, clinic site, primary language, education level, literacy, or access to healthcare provider (P < .0001)
**5**	Ornelas et al. 2018	USA Minority	Pretest and post-test survey design.	Type: E-health aid Duration: One day home visit Tools: 17 min Culturally tailored narrative video showed on iPad (n = 40)	Entertainment-education format: • Prologue: establishing the main characters and topic. • Core segments: focusing on logistic barriers to screening and screening procedures • Epilogue: closing the story and reminding viewers of key points. Characters: representing grandmother, mother & daughter.	Mean knowledge score (p<0.001) • Pre-test: 5.6 ±2.8 • Post-test: 9.3 ±1.0 Intention to CC screening • Pre-test: 40% Post-test: 100%
**6**	Thompson et al. 2019	USA Minority	A pilot randomized controlled design	Type: E-health aid Duration: One day survey (15–20 participants daily) within a 3-week window Tools: • IG: 5 min Digital Story showed on iPad (n = 42) • CG: Flu Fact Sheet (n = 42) *IG in this study also included: Fotonovela (n = 36) & Radionovela (n = 40). However, the result not been retrieved in this SLR.	Video of storytellers’ voices, music and pictures: • Characters: Latinas young woman, older sister, a mother & female doctor • The doctor explains the *human papillomavirus* and the HPV co-test using a plastic model of a woman’s reproductive system Ends with summary facts about HPV and the Pap test and encourages HPV vaccination for both girls and boys.	Mean knowledge score of CC risk factor (p = 0.02) • IG: 97.5 ±1.7 • CG: 85.5 ±4.2 Mean knowledge score of CC screening (p = 0.0003) • IG: 23.8 ±4.9 • CG: 13.7±3.3 Intention of CC screening (p = 0.06) • IG: 90.2% ±3.3 CG: 98.5% ±1.7

IG = intervention group; CG = control group

One study used a pilot randomized controlled design [11], two studies used a quasi-experimental design [40, 41] and three studies used a pre-test and post-test design [37–39]. Two studies used video entertainment educational aids as their e-health intervention [11, 37], two studies used movies [39, 40] and two study used video education aids [38, 41]. Four of the included studies mentioned the duration of the e-health intervention, which varied from 5 [11] to 25 minutes [40].

In total, 1300 women completed the e-health intervention and follow-ups. The sample sizes ranged from 40 [37] to 764 [39], with all studies investigating as their outcome knowledge improvement and either intention towards CC screening [11, 37] or CC screening uptake [38, 40].

### Intervention tool component regarding e-health

Table 2 summarises the components of the intervention tool, user involvement, outcome conclusion, eHealth Literacy Framework (eHLF) domains and quality ratings. Various approaches were used to implement e-health intervention, including entertainment education and narratives based on culturally tailored videos [37], the small media approach such as the digital story [11] and movie-based multimedia health education [40]. Community advisors and specific community users were involved in e-health tool development [11, 37]. All six studies used technology to process health information and three studies [11, 37, 40] included the understanding of health concepts and language in their e-health aids.

**Table 2:** Intervention tool’s component regarding e-health approach, users involvement, outcome conclusion, domains of the eHealth literacy framework and quality rating (n = 6).

Authors (Year)	E-health Approach	Users Involvement	Outcome conclusion	^a^eHLF		^b^EPHPP Quality Rating
				Using technology to process health information	Understanding of health concepts and language	
**Cooper et al. 2021**	Visual-audio methods of learning	Not mention	An effective, brief, practical with resource-appropriate teaching method may increase knowledge regardless of ultimate disease contraction, prior education and literacy level in the resource-limited settings.	√		Weak
**Nagamma et al. 2020**	Not mention	Not mention	The need for disseminating health information in the community and the importance of improving knowledge related to cervical cancer regardless of methods being used (audio-visual aid vs pamphlet).	√		Moderate
**Thompson et al. 2019**	Small media to reach specific audiences	FGD involve 23 Latinas.	Small media interventions (Digital Story, Fotonovela, Radionovela) using narrative education form are efficacious in changing knowledge and intention to receive pap testing	√	√	Strong
**Ornelas et al. 2018**	Entertainment-education & narratives based on culturally tailored videos	Community advisors	Participants’ suggestion: the video suitable using in a variety of settings and modalities such as clinic, community organization and mobile phone.	√	√	Weak
**Abiodun et al 2014**	Multimedia Health Education based on movie	Not mention	Knowledge and perception of CC and CC screening in rural communities improved by appropriate health education intervention.	√	√	Moderate
**Kessler et al. 2012**	Not mention	Not mention	The educational intervention was successful to increase knowledge of risks and screening guidelines in a 15-month period and better suited to younger women.	√		Weak

^a^eHLF = eHealth Literacy Framework

^b^EPHPP = Effective Public Health Practice Project Quality Assessment Tool

### Quality rating of included studies

Based on the EPHPP quality rating, one study was strong [11], two studies were moderate [40, 41] and three studies were weak [37–39]. Among the quality rating criteria, three studies received a double weak rating for selection bias because of their recruitment strategy and the assessor being aware of the participants’ status in either the intervention or the exposure group (S2 Table).

### Effectiveness of e-health intervention

#### Studies included in qualitative synthesis

**Fig 2:**
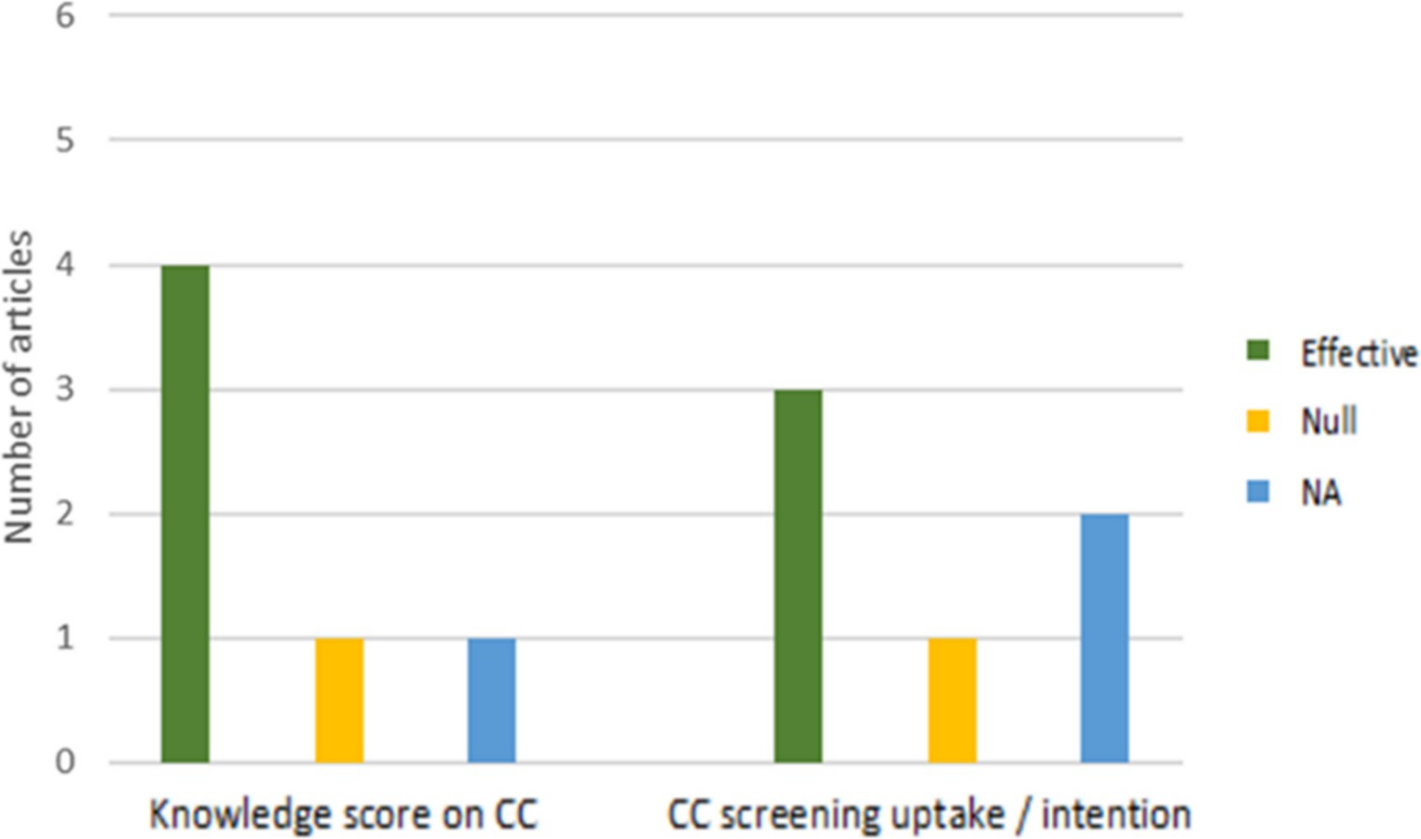
Comparison of effectiveness of e-health intervention on the knowledge score on CC and the uptake or intention of CC screening (n = 6). [NA = Data Not Available].

Examination of the effectiveness of e-health interventions (Fig 2) showed that four studies showed effectiveness of knowledge score regarding CC [11, 37, 39, 40]. The knowledge was assessed via pre-test and post-test questionnaire or survey. We only select the knowledge on CC to reviewed, as there was a diversity of knowledge measured by selected studies. Whereas, three studies showed increased intention [37] or uptake [38, 40] regarding CC screening. One study did not assess CC knowledge specifically [38] and one study did not evaluate the intention or uptake for CC screening [41]. Another study performed the screen-and-treat workshop as their intervention, thus the uptake was not calculated [39].

#### Studies included in the quantitative synthesis

We evaluated two outcomes for the meta-analysis: 1) The mean CC knowledge score, which involved four studies [11, 37, 39, 40]; and 2) The intention or uptake of CC screening, which involved four studies [11, 37, 38, 40]. One study [41] was excluded due to a lack of data.

**Fig 3:**
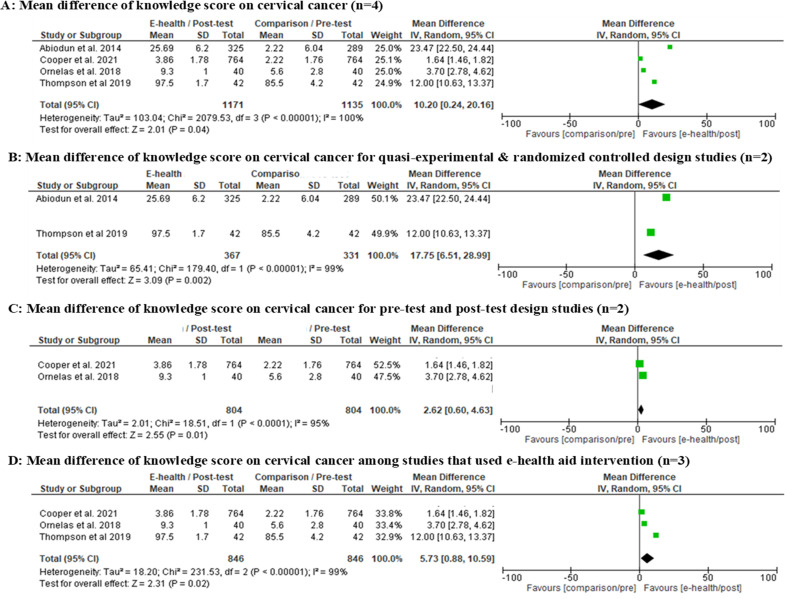
A: Random-effects forest plot for studies eligible for meta-analysis regarding mean difference knowledge score on cervical cancer (n = 4). B: Random-effects forest plot for studies that used quasi-experimental & randomized control design study (n = 2). C: Random effects forest plot for studies that used pre-test and post-test design study (n = 2). D: Random effects forest plot for studies that only used an e-health intervention aid (n = 3). Box size represents study weighting. Diamond represents overall effect size and 95% CI.

**Fig 4:**
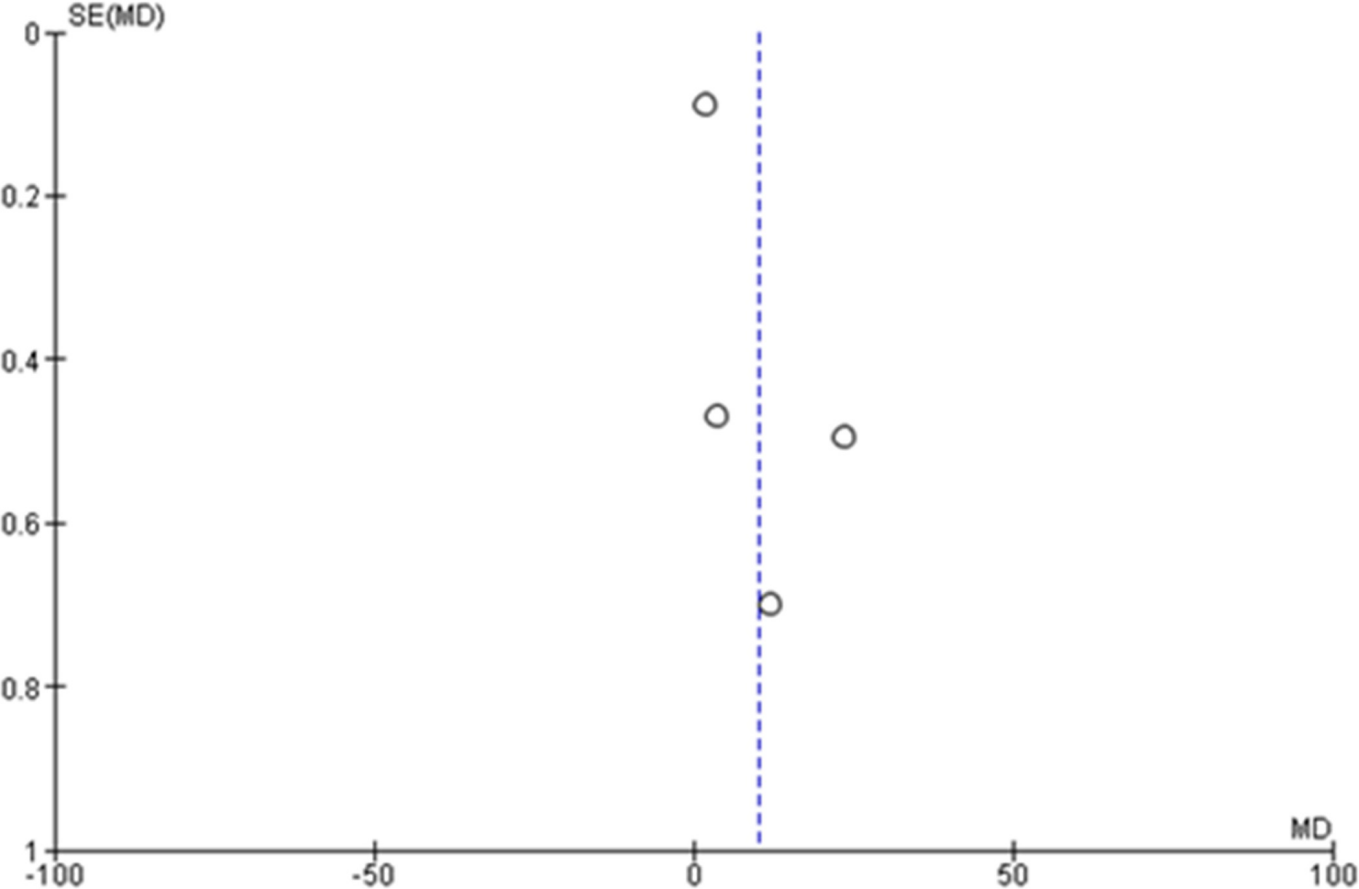
Funnel plot regarding mean knowledge score on cervical cancer for studies eligible for meta-analysis (n = 4).

A total of 2306 samples [study 1 [40] IG:n = 325, CG:n = 289 + study 2 [39] pre-test:n = 764, post-test:n = 764 + study 3 [37] pre-test:n = 40, post-test:n = 40 + study 4 [11] IG:n = 42; CG:n = 42] were pooled from four feasible studies for mean knowledge on CC regardless of their quality rating. The random-effects model revealed a statistically significant positive effect of the mean difference knowledge score using e-health intervention aids alone, where participants in the intervention groups were almost six time as likely to have increased knowledge on CC (mean difference = 5.73, 95% CI: 0.88–10.59, p < 0.05) (Fig 3). Nonetheless, clinical heterogeneity was high (I^2^ = 99%, p < 0.05) and the generated funnel plot was asymmetrical (Fig 4) due to differences among the studies. Whereas, the effect size for mean difference knowledge among selected studies range from 0.93 to 3.83 [study 1 [40] d = 3.83, 95% CI:3.56–4.09; study 2 [39] d = 0.93, 95% CI:0.82–1.03; study 3 [37] d = 1.76, 95% CI:1.24–2.27; study 4 [11] d = 3.74, 95% CI:3.03–4.45].

**Fig 5:**
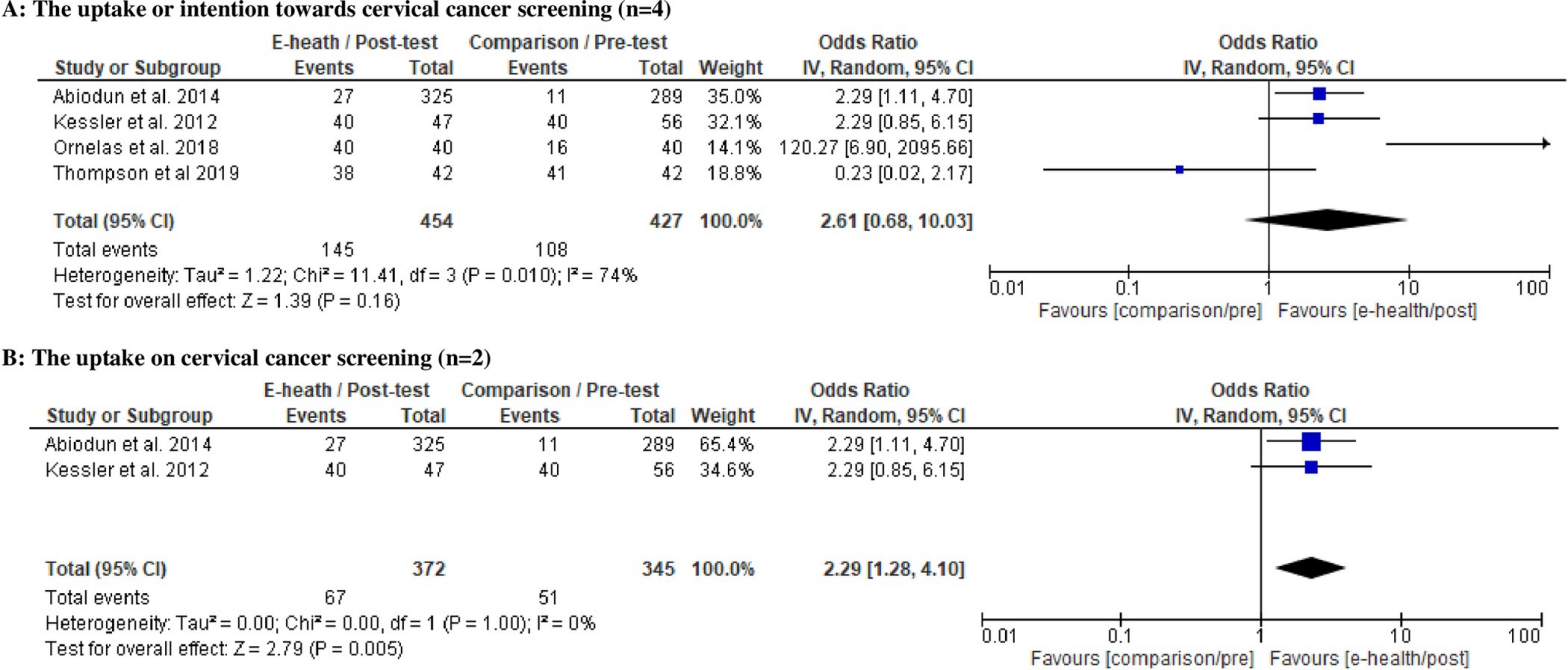
A: Random-effects forest plot for studies eligible for meta-analysis regarding the uptake or intention towards cervical cancer screening (n = 4). B: Random-effects forest plot for studies evaluated the uptake of cervical cancer screening (n = 2). Box size represents study weighting. Diamond represents overall effect size and 95% CI.

**Fig 6:**
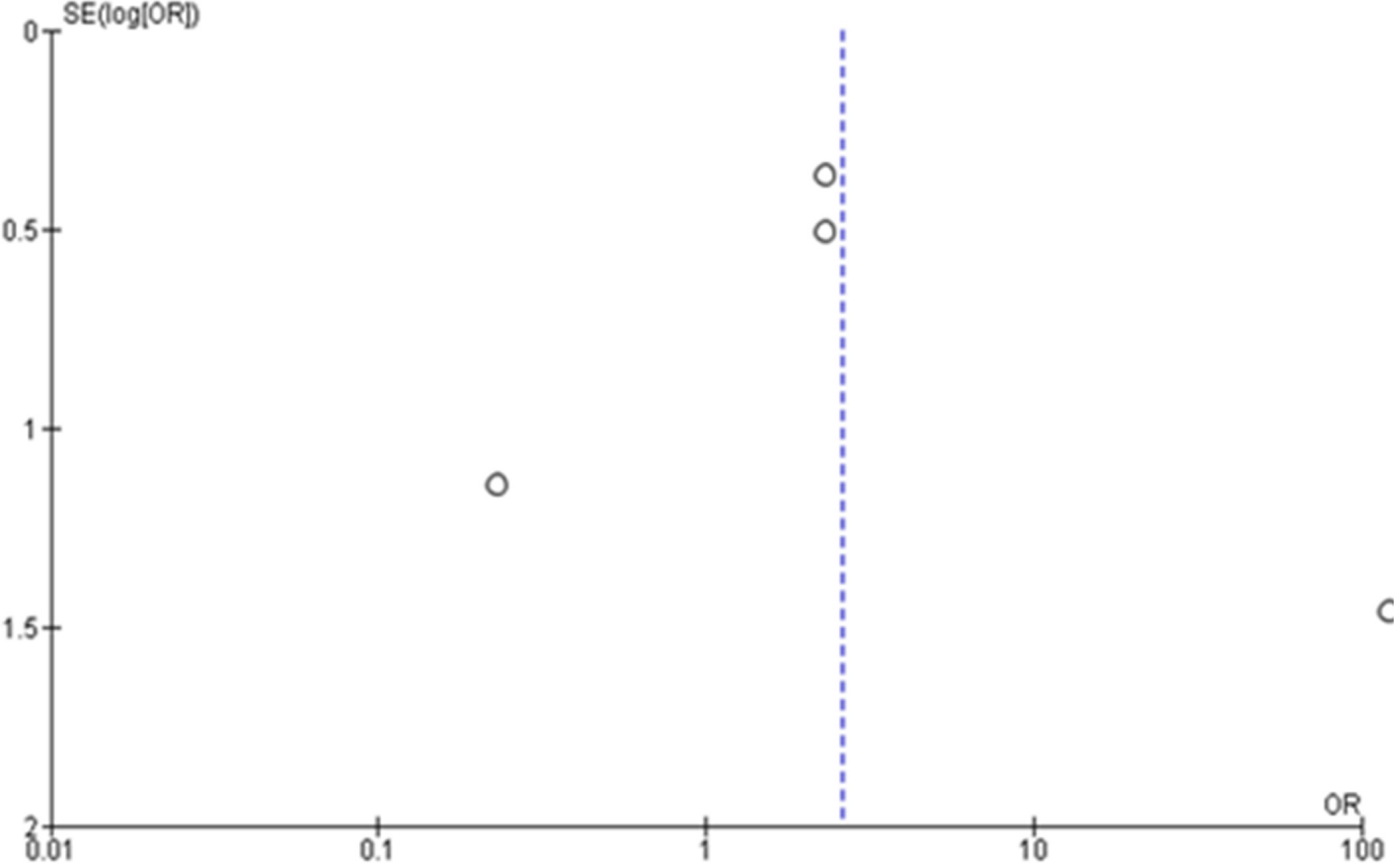
Funnel plot regarding the uptake or intention towards cervical cancer screening for studies eligible for meta-analysis (n = 4).

Meanwhile, a total of 881 samples [study 1 [40]; IG:n = 325, CG:n = 289 + study 2 [38] pre-test:n = 56, follow-up:n = 47 + study 3 [37] pre-test:n = 40, post-test:n = 40 + study 4 [11] IG:n = 42, CG:n = 42] were pooled for investigating the intention or uptake of CC screening. The random-effects model revealed a statistically significant positive effect on CC screening uptake using e-health interventions, where participants in the intervention groups were two-fold more likely to undergo CC screening (OR = 2.29, 95% CI: 1.28–4.10, p < 0.05) (Fig 5). Heterogeneity analyses in these subgroups yielded homogeneous results (I^2^ = 0%, p < 0.05). However, the funnel plot generated was asymmetrical (Fig 6) due to differences among the studies. Furthermore, the effect size of OR regarding the intention or uptake of CC screening among selected studies range from -0.810 to 2.641 [study 1 [40] d = 0.457; study 2 [38] d = 0.457; study 3 [37] d = 2.641; study 4 [11] d = -0.810].

## Discussion

### Principal findings

This systematic literature review presents an empirical investigation of the use of e-health interventions for CC and CC screening. Most of the included studies improved the knowledge on CC effectively and reached statistical significance (4/6), of which three studies used e-health aids alone through video entertainment education [11, 37] or movies [39]. Although knowledge enhancement was effective, the methodological quality was low [37, 39]. Thus, the effectiveness might be insufficiently strong due to the study using the pre-test and post-test design. Meanwhile, a study that used a 5-minute digital story with a strong quality rating [11] proved that using female characters in various age groups can impart knowledge to the targeted audience.

Two studies improved CC screening uptake effectively [38, 40]. Both studies used a mixed-education method of e-health with didactic sessions, where uptake was measured at the 15-month [38] and 13-week [40] follow-up. Women with adequate health information along with self-efficacy would undergo screening when a given follow-up period is adequate. However, both of these studies had low quality ratings due to the use of the pre-test and post-test design [38] and a quasi-experimental study [40]. The random-effects forest plot showed high clinical heterogeneity for CC knowledge (I^2^ = 99%, p < 0.05) and CC screening uptake or intention (I^2^ = 74%, p < 0.05) with an asymmetrical funnel plot. These results may show possible bias due to the varied study designs, population groups, sample sizes, e-health aid content and durations.

Since the high heterogeneity were found, the effect size was calculated to show clinically relevant effect for future practise. The effect size ranging from medium (d = 0.457 for two studies on CC screening uptake) to large (range of d = 0.93–3.83 for four studies on mean difference in CC knowledge). Therefore, even though the number of studies included is small with high heterogeneity, the effect size still showed superior and clinically relevant to use e-health intervention for improving CC knowledge and CC screening uptake.

### The need for framework integration into e-health intervention

Most of the included studies mentioned the e-health approach used in implementing their intervention. However, the use of a theoretical framework was not specified. Only two studies mentioned the framework used as guidelines in developing the e-health interventions, which was the behavioural model for vulnerable populations [37] and Bandura’s (1986) concept of self-efficacy [38]. Studies which used a framework demonstrated an improvement in participant knowledge [37] and screening uptake [38]. The video that used the behavioural model for vulnerable populations is suitable for use in a variety of settings and modalities such as clinics, community organisations and through mobile phones [37]. These two studies represent the developed nations, with high readiness of e-health implementation.

On the other hand, implementation of e-health interventions in the developing countries and LMICs may be achieved with organisational and technological infrastructural readiness along with public-patient readiness [42]. A recent review identified a similar achievement between audio-visual aids integrated into a 30-minute teaching intervention and pamphlet intervention in a rural setting [41]. Meanwhile, a 15-minute Medical Aid film alone significantly improved participants’ knowledge regardless of age group, clinic site, primary language, education level, literacy or access to healthcare provider (p < 0.0001) [39]. A longer duration of health education sessions does not guarantee understanding. E-interventions alone that are engaging and easy to understand are more effective. However, both studies were implemented in a community setting; hence, the technology infrastructure readiness could not be assessed.

Furthermore, all included studies used technology to process health information as an eHLF-derived domain. The intervention components contain information on CC and understanding CC screening, treatment and prevention. The eHLF domains may provide new insight into the ability to understand, access and use e-health technology [43]. The other eHLF domains, namely understanding health concepts and language, could be identified by involving users in the development of e-health aids [11, 37]. WHO has recommended the involvement of women as potential users of e-health aid in CC screening and empowered women to lead the development of material to make e-health aid accessible and useable [8]. Thus, the infusion of narratives based on cultural tailoring also helps to enhance the positive effect of health interventions.

### Implication and potential of e-health aid for public health

The current coronavirus disease 2019 (COVID-19) global pandemic will not stop health information dissemination through e-health aids. In fact, e-health has been a major focus of the WHO since 2005, followed by the resurgence of the use of health-focused technology by the developed countries beginning in 2006 [23, 42]. Towards the 21st century, the WHO presented a global strategy towards eliminating CC from public health concern. The vision of the strategy guidelines included a threshold rate of ASR 4.0 per 100 000 women with 70% of women screened before the age of 35 years and re-screened by 45 years [8]. Hence, countries with a higher ASR need to move into e-health promotion as a proactive step towards reaching the unreached. Health information through electronic play role in reinforcing positive health behaviours especially among youngest generation [44]. The world is heading for a decade’s effort towards the WHO Shanghai Declaration on promoting health in the 2030 Agenda and on the full use of social innovation and interactive technology towards prioritizing health policy [45]. E-health has gained attention worldwide and has eliminated geographic barriers [46].

### Limitations

Although we have demonstrated the positive effects of e-health intervention in our review, the reporting, publication bias and low quality rating may have affected our findings. The effect of the mixed-education method could have influenced e-health intervention through the varied settings with multiple study designs, as shown by the high heterogeneity. Furthermore, half of the included studies were low quality due to the pre-test and post-test design. Thus, the effectiveness result might be compromised.

## Conclusion

This review demonstrates the effectiveness of e-health interventions for enhancing knowledge, intention or uptake of CC screening. It proves that electronics are easy tools for reaching urban, rural and minority communities worldwide. To achieve the WHO goal of eliminating CC as a public health problem, policymakers need to start reaching out to the marginalized through e-health interventions. The use of appropriate frameworks, user engagement and culturally tailored e-health needs to be prioritised. However, due to high heterogeneity and lack of study in this review, we encourage further empirical investigation regarding e-health aids in future.

## Supporting information

S1 Checklist PRISMA 2020 checklist. (DOCX)

S1 Table Search strategy and MeSH keywords used. (DOCX)

S2 Table Risk of bias assessment according to the EPHPP. (DOCX)

S3 Table Full search string strategies. (DOCX)

## References

[1] Cohen P.A., et al., Cervical cancer. The Lancet, 2019. 393(10167): p. 169–182. 10.1016/S0140-6736(18)32470-X 30638582

[2] Smith R.A., et al., Cancer screening in the United States, 2019: a review of current American Cancer Society guidelines and current issues in cancer screening. CA: a cancer journal for clinicians, 2019. 69(3): p. 184–210. doi: 10.3322/caac.21557 30875085

[3] Chew K.T., Kampan N., and Shafiee M.N., Perception and knowledge of human papillomavirus (HPV) vaccine for cervical cancer prevention among fully vaccinated female university students in the era of HPV vaccination: a cross-sectional study. BMJ open, 2021. 11(12): p. e047479. doi: 10.1136/bmjopen-2020-047479 34876417 PMC8655553

[4] Gönenç İ.M., et al., A review of knowledge and attitudes of young people on cervical cancer and HPV vaccination. Journal of Public Health, 2020. 28(1): p. 97–103.

[5] Wanderley M.d.S, et al., Students’ HPV vaccination rates are associated with demographics, sexuality, and source of advice but not level of study in medical school. Revista do Instituto de Medicina Tropical de São Paulo, 2019. 61. 10.1590/S1678-9946201961070 PMC6922017 31859847

[6] Fisher W.A., et al., Barriers to human papillomavirus vaccine acceptability in Israel. Vaccine, 2013. 31: p. I53–I57. doi: 10.1016/j.vaccine.2013.06.107 24229720

[7] Sundström K. and Elfström K.M., Advances in cervical cancer prevention: Efficacy, effectiveness, elimination? 2020, Public Library of Science San Francisco, CA USA. p. e1003035. 10.1371/journal.pmed.1003035 PMC6986699 31990905

[8] World Health Organization. Draft global strategy towards eliminating cervical cancer as a public health problem. 2020. 2020 5 April 2020 [cited 2021 30 September 2021]; Available from: https://www.who.int/publications/m/item/draft-global-strategy-towards-eliminating-cervical-cancer-as-a-public-health-problem.

[9] Curry S.J., et al., Screening for cervical cancer: US Preventive Services Task Force recommendation statement. Jama, 2018. 320(7): p. 674–686. doi: 10.1001/jama.2018.10897 30140884

[10] Wearn A. and Shepherd L., The impact of emotion-based mass media campaigns on stigma toward cervical screening non participation. Journal of Applied Social Psychology, 2020. 50(5): p. 289–298.

[11] Thompson B., et al., Educating Latinas about cervical cancer and HPV: a pilot randomized study. Cancer causes & control: CCC, 2019. 30(4): p. 375–384. doi: 10.1007/s10552-019-01150-w 30830494 PMC6459715

[12] Lyson H.C., et al., Social Media as a Tool to Promote Health Awareness: Results from an Online Cervical Cancer Prevention Study. Journal of cancer education: the official journal of the American Association for Cancer Education, 2019. 34(4): p. 819–822. doi: 10.1007/s13187-018-1379-8 29948924 PMC6289895

[13] Park H.G., et al., The association between social media use for health related information and compliance with breast and cervical cancer screenings. Research reports (Montgomery), 2020. 4: p. e1–e14. 34278179 PMC8281882

[14] Kelly J.T., et al., The Internet of Things: Impact and implications for health care delivery. Journal of medical Internet research, 2020. 22(11): p. e20135. doi: 10.2196/20135 33170132 PMC7685921

[15] Lewis J., Ray P., and Liaw S.-T., Recent worldwide developments in eHealth and mHealth to more effectively manage cancer and other chronic diseases–a systematic review. Yearbook of medical informatics, 2016. 25(01): p. 93–108. doi: 10.15265/IY-2016-020 27830236 PMC5171554

[16] Cheng C., et al., Applying the electronic health literacy lens: systematic review of electronic health interventions targeted at socially disadvantaged groups. Journal of medical Internet research, 2020. 22(8): p. e18476. doi: 10.2196/18476 32788144 PMC7453328

[17] Griebel L., et al., eHealth literacy research—Quo vadis? Informatics for Health and Social Care, 2018. 43(4): p. 427–442. doi: 10.1080/17538157.2017.1364247 29045164

[18] Page M.J., et al., The PRISMA 2020 statement: an updated guideline for reporting systematic reviews. Bmj, 2021. 372. 10.1136/bmj.n71 PMC8005924 33782057

[19] Arbyn M., et al., Estimates of incidence and mortality of cervical cancer in 2018: a worldwide analysis. The Lancet Global Health, 2020. 8(2): p. e191–e203. doi: 10.1016/S2214-109X(19)30482-6 31812369 PMC7025157

[20] Moreno-Guerrero A.-J., et al., Internet Addiction in the Web of Science Database: A Review of the Literature with Scientific Mapping. International Journal of Environmental Research and Public Health, 2020. 17(8): p. 2753. doi: 10.3390/ijerph17082753 32316177 PMC7216291

[21] Basson I., Blanckenberg J.P., and Prozesky H., Do open access journal articles experience a citation advantage? Results and methodological reflections of an application of multiple measures to an analysis by WoS subject areas. Scientometrics, 2021. 126(1): p. 459–484.

[22] Falagas M.E., et al., Comparison of PubMed, Scopus, web of science, and Google scholar: strengths and weaknesses. The FASEB journal, 2008. 22(2): p. 338–342. doi: 10.1096/fj.07-9492LSF 17884971

[23] Paige S.R., et al., Electronic health literacy across the lifespan: measurement invariance study. Journal of medical Internet research, 2018. 20(7): p. e10434. doi: 10.2196/10434 29986848 PMC6056742

[24] Hewapathirana R., et al., ‘Hybrid Doctors’ Can Fast Track the Evolution of a Sustainable e-Health Ecosystem in Low Resource Contexts: The Sri Lankan Experience. Studies in Health Technology and Informatics, 2019. 264: p. 1356–1360. doi: 10.3233/SHTI190448 31438147

[25] Green M.J. and Levi B.H., The era of “e”: the use of new technologies in advance care planning. Nursing outlook, 2012. 60(6): p. 376–383. e2. doi: 10.1016/j.outlook.2012.08.005 23141197 PMC3548399

[26] Muljo H.H., et al., Mobile learning for early detection cancer. International Journal of Interactive Mobile Technologies, 2018. 12(2): p. 39–53.

[27] Duong H.T. and Hopfer S., "Let’s Chat": Process evaluation of an intergenerational group chat intervention to increase cancer prevention screening among Vietnamese American families. Translational Behavioral Medicine, 2021. 11(3): p. 891–900. doi: 10.1093/tbm/ibaa120 33290557 PMC12622224

[28] Thomas B., et al., A process for systematically reviewing the literature: providing the research evidence for public health nursing interventions. Worldviews on Evidence‐Based Nursing, 2004. 1(3): p. 176–184. doi: 10.1111/j.1524-475X.2004.04006.x 17163895

[29] Armijo‐Olivo S., et al., Assessment of study quality for systematic reviews: a comparison of the Cochrane Collaboration Risk of Bias Tool and the Effective Public Health Practice Project Quality Assessment Tool: methodological research. Journal of evaluation in clinical practice, 2012. 18(1): p. 12–18. doi: 10.1111/j.1365-2753.2010.01516.x 20698919

[30] RevMan, Review Manager (RevMan). 2020, The Cochrane Collaboration.

[31] Chinn S., A simple method for converting an odds ratio to effect size for use in meta‐analysis. Statistics in medicine, 2000. 19(22): p. 3127–3131. doi: 10.1002/1097-0258(20001130)19:22&lt;3127::aid-sim784&gt;3.0.co;2-m 11113947

[32] Ferguson C.J., An effect size primer: a guide for clinicians and researchers. 2016.

[33] Lin H. Convert between different effect sizes. 2022 [cited 2022 30.3.2022]; Available from: https://www.escal.site/.

[34] Higgins J.P., et al., Measuring inconsistency in meta-analyses. Bmj, 2003. 327(7414): p. 557–560. doi: 10.1136/bmj.327.7414.557 12958120 PMC192859

[35] Sterne J.A. and Harbord R.M., Funnel plots in meta-analysis. The stata journal, 2004. 4(2): p. 127–141.

[36] GLOBOCAN, Estimated age-standardized incidence rates (World) in 2020, cervix uteri, females, all ages, Observatory G.C., Editor. 2022, International Agency for Research on Cancer, World Health Organization.

[37] Ornelas I.J., et al., Results from a pilot video intervention to increase cervical cancer screening in refugee women. Health Education & Behavior, 2018. 45(4): p. 559–568. doi: 10.1177/1090198117742153 29202606 PMC7012240

[38] Kessler T.A. Increasing mammography and cervical cancer knowledge and screening behaviors with an educational program. in Oncology Nursing Forum. 2012. doi: 10.1188/12.ONF.61-68 22201656

[39] Cooper E.C., et al., Implementation of human papillomavirus video education for women participating in mass cervical cancer screening in Tanzania. American journal of obstetrics and gynecology, 2021. 224(1): p. 105. e1–105. e9. doi: 10.1016/j.ajog.2020.07.018 32682861

[40] Abiodun O.A., et al., Impact of health education intervention on knowledge and perception of cervical cancer and cervical screening uptake among adult women in rural communities in Nigeria. Bmc Public Health, 2014. 14. doi: 10.1186/1471-2458-14-814 25103189 PMC4133628

[41] Nagamma T., et al., Effectiveness of audio-visual and print media intervention on knowledge of cervical health among rural women in Southern India. The Nigerian postgraduate medical journal, 2020. 27(4): p. 343–347. doi: 10.4103/npmj.npmj_148_20 33154288

[42] Mauco K.L., Scott R.E., and Mars M., Development of a Conceptual Framework for e-Health Readiness Assessment in the Context of Developing Countries, in Telehealth Innovations in Remote Healthcare Services Delivery. 2021, IOS Press. p. 68–77.

[43] Norgaard O., et al., The e-health literacy framework: a conceptual framework for characterizing e-health users and their interaction with e-health systems. Knowledge Management & E-Learning: An International Journal, 2015. 7(4): p. 522–540.

[44] Shinta A.D., Salleh M.A.M., and Ali M.N.S., Analysis of the Moderating Effect of Media Literacy on Cervical Cancer Preventive Behaviours. Jurnal Komunikasi-Malaysian Journal of Communication, 2019. 35(1): p. 156–170.

[45] World Health Organization, Shanghai Declaration on Promoting Health in the 2030 Agenda for Sustainable Development 2016. Available online: Accessed, 2017. 23. 10.1093/heapro/daw103 28180270

[46] Biancone P., et al., E-health for the future. Managerial perspectives using a multiple case study approach. Technovation, 2021: p. 102406.

