# Supplementary material for: Empirical investigation of e-health intervention in cervical cancer screening: A systematic literature review
Source: PLoS One. 2022 Aug 19;17(8):e0273375. doi: 10.1371/journal.pone.0273375 (PMC9390916; doi:10.1371/journal.pone.0273375)
Supplement: S1 Table — (DOCX) [file pone.0273375.s002.docx]

S1 Table: Search strategy and the medical subject heading (MeSH) keywords used.

| Number | Search term |
| --- | --- |
| Database used: Web Of Science (WOS), Scopus, EBSCO Medline Complete via Medical Databases | |
| 1 | "cervical cancer" OR "uterine cervical neoplasms" OR "uterine cervical carcinoma" OR "cervical tum?r" OR "cervical malignancy" OR "cervix tum?r" OR "cervix malignancy" OR "cervix cancer" OR "cervical neck tum?r" OR "cervical neck malignancy" OR "cervical neck cancer" OR "uterine cervix cancer" OR "uterine cervix tum?r" OR "uterine cervix malignancy" OR "cervix uteri cancer" OR "cervix uteri malignancy" OR "cervix uteri tum?r" |
| 2 | "screening" OR "papanicolaou test" OR "papanicolaou smear" OR "pap smear" OR "pap test" OR "human papillomavirus DNA tests" OR "HPV DNA tests" OR "human papillomavirus test" OR "HPV test" |
| 3 | 1 OR 2 |
| 4 | "electronic health" OR "electronic health intervention" OR "e-health" OR "video education" OR "informative video" OR "media" OR "digital media" OR "digital literacy" |
| 5 | "screening uptake" OR "screening take-up" OR "practice" OR "perform" OR "participate" |
| 6 | "knowledge" OR "awareness" OR "educate*" OR "understand*" OR "insight" OR "percept*" OR "belief*" OR "learn*" |
| 7 | "motivation" OR "motive*" OR "induce*" OR "expectation" OR "incentive" OR "aspiration" OR "drive" OR "instinct" OR "intention" OR "empowerment" OR "catalyst" OR "desire" OR "encourage*" OR "impulse*" |
| 8 | 3 AND 4 AND 5 |
| 9 | 3 AND 4 AND 6 |
| 10 | 3 AND 4 AND 7 |
