# Supplementary material for: Empirical investigation of e-health intervention in cervical cancer screening: A systematic literature review
Source: PLoS One. 2022 Aug 19;17(8):e0273375. doi: 10.1371/journal.pone.0273375 (PMC9390916; doi:10.1371/journal.pone.0273375)
Supplement: S2 Table — (DOCX) [file pone.0273375.s003.docx]

S2 Table: Risk of bias assessment according to the Effective Public Health Practice Project Quality Assessment Tool (EPHPP).

| Authors (Year) | Selection Bias | Study Design | Confounders | Blinding | Data Collection Methods | Withdrawals and Drop-outs | Global Rating |
| --- | --- | --- | --- | --- | --- | --- | --- |
| Nagamma et al. 2021 | moderate | strong | strong | weak | strong | strong | moderate |
| Cooper et al. 2020 | weak | moderate | weak | weak | strong | strong | weak |
| Ornelas et al. 2018 | weak | moderate | weak | weak | strong | strong | weak |
| Thompson et al. 2018 | moderate | strong | strong | strong | strong | strong | strong |
| Abiodun et al. 2014 | moderate | strong | strong | weak | strong | strong | moderate |
| Kessler et al. 2012 | weak | moderate | weak | weak | strong | strong | weak |
