# Supplementary material for: Empirical investigation of e-health intervention in cervical cancer screening: A systematic literature review
Source: PLoS One. 2022 Aug 19;17(8):e0273375. doi: 10.1371/journal.pone.0273375 (PMC9390916; doi:10.1371/journal.pone.0273375)
Supplement: S3 Table — (DOCX) [file pone.0273375.s004.docx]

Title: Empirical Investigation of E-Health Intervention in Cervical Cancer Screening:

A Systematic Review.

S3 Table: Full search string strategies.

| Web Of Science  (WOS) | Identification  (120 article) | “Cervical cancer” OR “Uterine Cervical Neoplasms” OR “uterine Cervical carcinoma” OR “cervical tum?r” OR “cervical malignancy” OR “cervix tum?r” OR ”cervix malignancy” OR “cervix cancer” OR “cervical neck tum?r” OR “cervical neck malignancy” OR “cervical neck cancer” OR “Uterine cervix cancer” OR “Uterine cervix tum?r” OR “Uterine cervix malignancy” OR “Cervix uteri cancer” OR “Cervix uteri malignancy” OR “Cervix uteri tum?r” AND “Screening” OR “Papanicolaou test” OR “Papanicolaou smear” OR “Pap smear” OR “Pap test” OR “Human Papillomavirus DNA Tests” OR “HPV DNA Tests” OR “Human papillomavirus test” OR “HPV test” (Title) and “electronic health” OR “electronic health intervention” OR “e-health” OR “video education” OR “informative video” OR “media” OR “Digital media” OR “digital literacy” (Abstract) and “screening uptake” OR “screening take-up” OR “practice” OR “perform” OR “participate” OR “knowledge” OR “awareness” OR “educate*” OR “understand*” OR “insight” OR “percept*” OR “belief*” OR “learn*” OR “Motivation” OR “motive*” OR “induce*” OR “Expectation” OR “Incentive” OR “Aspiration” OR “Drive” OR “Instinct” OR “Intention” OR “Empowerment” OR “Catalyst” OR “Desire” OR “Encourage*” OR “Impulse*” |
| --- | --- | --- |
|  | Screening  (86 article) | Limit to =   1. Year (2011-2021) – 94 2. Document type (Articles) – 90 3. English – 86 |
|  | Eligibility  (4 article) | 1. Title 2. Abstract |
| Scopus | Identification  (505 article) | ( TITLE-ABS-KEY ( "Cervical cancer" OR "Uterine Cervical Neoplasms" OR "uterine Cervical carcinoma" OR "cervical tum?r" OR "cervical malignancy" OR "cervix tum?r" OR "cervix malignancy" OR "cervix cancer" OR "cervical neck tum?r" OR "cervical neck malignancy" OR "cervical neck cancer" OR "Uterine cervix cancer" OR "Uterine cervix tum?r" OR "Uterine cervix malignancy" OR "Cervix uteri cancer" OR "Cervix uteri malignancy" OR "Cervix uteri tum?r" AND "Screening" OR "Papanicolaou test" OR "Papanicolaou smear" OR "Pap smear" OR "Pap test" OR "Human Papillomavirus DNA Tests" OR "HPV DNA Tests" OR "Human papillomavirus test" OR "HPV test" ) AND TITLE-ABS-KEY ( "electronic health" OR "electronic health intervention" OR "e-health" OR "video education" OR "informative video" OR "media" OR "Digital media" OR "digital literacy" ) AND TITLE-ABS-KEY ( "screening uptake" OR "screening take-up" OR "practice" OR "perform" OR "participate" OR "knowledge" OR "awareness" OR "educate*" OR "understand*" OR "insight" OR "percept*" OR "belief*" OR "learn*" OR "Motivation" OR "motive*" OR "induce*" OR "Expectation" OR "Incentive" OR "Aspiration" OR "Drive" OR "Instinct" OR "Intention" OR "Empowerment" OR "Catalyst" OR "Desire" OR "Encourage*" OR "Impulse*" ) |
|  | Screening  (302 article) | Limit to =   1. Year (2011-2021) – 368 2. Document type (article) – 315 3. English – 302 |
|  | Eligibility  (8 article) | 1. Title 2. Abstract |
| EBSCO Medline Complete  via  Medical Databases | Identification  (231 article) | TI ( “Cervical cancer” OR “Uterine Cervical Neoplasms” OR “uterine Cervical carcinoma” OR “cervical tum?r” OR “cervical malignancy” OR “cervix tum?r” OR ”cervix malignancy” OR “cervix cancer” OR “cervical neck tum?r” OR “cervical neck malignancy” OR “cervical neck cancer” OR “Uterine cervix cancer” OR “Uterine cervix tum?r” OR “Uterine cervix malignancy” OR “Cervix uteri cancer” OR “Cervix uteri malignancy” OR “Cervix uteri tum?r” AND “Screening” OR “Papanicolaou test” OR “Papanicolaou smear” OR “Pap smear” OR “Pap test” OR “Human Papillomavirus DNA Tests” OR “HPV DNA Tests” OR “Human papillomavirus test” OR “HPV test” ) AND AB ( “electronic health” OR “electronic health intervention” OR “e-health” OR “video education” OR “informative video” OR “media” OR “Digital media” OR “digital literacy” ) AND AB ( “screening uptake” OR “screening take-up” OR “practice” OR “perform” OR “participate” OR “knowledge” OR “awareness” OR “educate*” OR “understand*” OR “insight” OR “percept*” OR “belief*” OR “learn*” OR “Motivation” OR “motive*” OR “induce*” OR “Expectation” OR “Incentive” OR “Aspiration” OR “Drive” OR “Instinct” OR “Intention” OR “Empowerment” OR “Catalyst” OR “Desire” OR “Encourage*” OR “Impulse*” ) |
|  | Screening  (107 article) | Limit to =   1. Year (2011-2021) – 171 2. Document type (Journals) – 166 3. English – 159 4. Duplicate removed intra database – 107 |
|  | Eligibility  (6 article) | 1. Title 2. Abstract |
| Reference Tracking | Eligibility  (6 article) | 1. Title 2. Abstract |
